# Role of miRNA in Melanoma Development and Progression

**DOI:** 10.3390/ijms24010201

**Published:** 2022-12-22

**Authors:** Agata Poniewierska-Baran, Sylwia Słuczanowska-Głąbowska, Paulina Małkowska, Olga Sierawska, Łukasz Zadroga, Andrzej Pawlik, Paulina Niedźwiedzka-Rystwej

**Affiliations:** 1 Institute of Biology, University of Szczecin, 71-412 Szczecin, Poland; 2 Department of Physiology, Pomeranian Medical University in Szczecin, 70-111 Szczecin, Poland; 3 Doctoral School of the University of Szczecin, Institute of Biology, University of Szczecin, 71-412 Szczecin, Poland; 4 Pomeranian Medical University in Szczecin, 70-111 Szczecin, Poland

**Keywords:** miRNA, microRNA, melanoma, skin cancer, melanoma metastasis, melanoma progression

## Abstract

Melanoma is one of the most aggressive and progressive skin cancers. It develops from normal pigment-producing cells known as melanocytes, so it is important to know the mechanism behind such transformations. The study of metastasis mechanisms is crucial for a better understanding the biology of neoplastic cells. Metastasis of melanoma, or any type of cancer, is a multi-stage process in which the neoplastic cells leave the primary tumour, travel through the blood and/or lymphatic vessels, settle in distant organs and create secondary tumours. MicroRNA (miRNA) can participate in several steps of the metastatic process. This review presents the role of miRNA molecules in the development and progression as well as the immune response to melanoma.

## 1. Introduction

Melanoma is the most serious type of skin cancer and causes a large number of deaths. The metastasis process consists of many stages, including the proliferation of cells within the forming tumour, the proteolytic activity of cancer cells, their ability to migrate and adhere and the formation of blood vessels supplying newly formed tumours. Profiling of miRNA expression in melanocytes as well as melanoma cells originating from primary vs. metastatic cells can provide valuable data about the role of specific miRNA in each phase of cancer development, focusing on the tumour microenvironment—interactions with cancer-associated fibroblasts (CAFs), tumour-associated macrophages (TAMs), mesenchymal stem cells (MSCs), endothelial cells (ECs) and T cells—and tumour progression based on growth, angiogenesis, metastasis, drug resistance and immune response to cancer. As will be presented in this review, miRNAs are involved in all stages of metastasis in melanoma, from the initiation of the angiogenesis process to the formation of secondary tumours in distant organs.

## 2. MiRNA in the Tumour Microenvironment

The tumour microenvironment (TME) plays an important role in tumour development, progression as well as tumour response to therapy. Therefore, tumour development depends not only on the presence of cancer cells but also on the microenvironment in which these cells grow [1]. Cancer is a natural response to an abnormal stromal environment [2]. Similarities between carcinogenesis and wound healing suggest that cancers can be viewed as ‘wounds that do not heal’ [3,4]. The tumour microenvironment is made up of many components, including endothelial cells, pericytes (PCs), fibroblasts (Fbs), inflammatory cells (e.g., T cells and macrophages), tumour-associated macrophages, cancer-associated fibroblasts and pro-inflammatory molecules secreted by these cells, such as interleukins (e.g., IL-6), chemokines (e.g., CXCL12/SDF-1α), vascular endothelial growth factors (VEGFs) and platelet-derived growth factors (PDGFs), matrix metalloproteinases (MMPs) and components of the extracellular matrix (ECM; e.g., tenascin C, fibronectin and collagen type I). Cytokines secreted by various cell types recruit bone marrow-derived cells and immune system cells into the vicinity. Interestingly, experimental studies have shown that the TME, under certain conditions, can restore cancer cells to normal cells by altering specific expression of miRNA [5], indicating that stromal TME cells can be a potential effective cancer treatment strategy [1]. These cells can significantly contribute to metastasis and tumour progression via deregulation of miRNA expression or activity. It has been shown that miRNAs are essential players in the crosstalk between cancer cells and the TME [6,7,8].

### 2.1. Role of miRNA in Regulating Cancer-Associated Fibroblasts (CAFs)

Fibroblasts are major components of the TME [9]. In the early stages of cancer formation, normal fibroblasts can stop tumour growth and migration [10], but CAFs work in the opposite manner, as they create an environment that promotes tumour formation and progression [11,12,13]. CAFs promote blood vessel formation (angiogenesis) by ECs and pericytes to facilitate cancer cell metastasis, so they represent an important target for anti-cancer therapies [14]. CAFs, compared to Fbs, present high expression of smooth muscle α-actin and secrete pro-inflammatory molecules that promote tumour growth; thus, CAFs can modulate inflammation in the vicinity of the tumour and promote communication between cells [14].

It has been shown that miRNA regulate the CAF phenotype in many types of cancer, i.e., gastric [15], pancreatic [16], colorectal [17], hepatocellular [18], breast [19], head and neck cancer [20], as well as in melanoma [21]. Melanocytes cells are able to product pigment vesicles called melanosomes, which carry miRNA (e.g., miR-211) into primary fibroblasts. This results in increased proliferation and migration of cells, as well as expression of pro-inflammatory genes, exactly how CAFs behave [22]. Interestingly, inhibiting melanosome release can prevent CAF formation, but at the same time, melanoma cells and CAFs release exosomal miRNA, which promotes CAF formation. By using pre- or anti-miRNA inhibitors to reconstitute the unnatural expression of miRNA in CAF cells, Shen et al. (2016) inhibited proliferation and development of cancer cells in mouse models [23]. Pro-cancerous miR-21, miR-31, miR-214 and miR-155 appear to play roles in the differentiation of normal fibroblasts to CAFs, depending on the type of tumour [24].

### 2.2. Role of miRNA in Regulating T Cells

T cell maturation, activation and function depend on miRNA. Immuno-suppressive FOXP3+ regulatory T cells (Tregs) are present in tumour tissues, and they influence cancer growth and progression [25]. Tumour-secreted miR-214 mobilises Tregs to release higher levels of IL-10 by downregulating PTEN, leading to immune-suppression and rapid tumour growth. Blocking tumour-secreted miR-214 inhibits the action of Tregs and tumour progression, indicating a potential treatment target of limiting Treg expansion and tumour growth [25]. It has been reported that silencing of miR-21 reduces the proliferation of Tregs (by PTEN), followed by activation of the Akt pathway. Another silencing miRNA molecule that influences Treg expression of FOXP3 is miR-126 [26]; in a melanoma mouse model, silencing of miR-126 in Tregs activated the anti-tumour response of CD8^+^ T cells.

When it comes to studies conducted on T cells in the melanoma microenvironment, interesting results have been described [27,28,29,30]. Maibach et al. (2020) described that the presence of lymphocytes in the melanoma tumour mass is usually an optimistic prognostic factor, but it depends on which population of T cells is detected, i.e., the presence of T lymphocytes (CD3^+^, CD8^+^, CD4^+^) and B lymphocytes (CD20^+^) correlates with a better prognosis for the patient, but Tregs (FOXP3^+^) are associated with a worse prognosis [30]. In 2020, Tittarelli et al. published results showing that miRNA is released from hypoxic melanoma cells to dendritic cells (DCs) and melanoma-specific cytotoxic T (Tc) cells [27]. They identified that miR-192-5p was strongly secreted by melanoma cells under hypoxic conditions, which then downregulated the activity of melanoma-specific cytotoxic T lymphocytes (resistance strategy). Norman et al. (2012) showed how miR-210 mediates immunosuppression in oxygen-deprived regions of the tumour (hypoxia), where cancer stem-like cells and metastatic behaviours evolve [29]. It has been shown that the expression of 70 different miRNAs depends on oxygen conditions (hypoxia or normoxia) and that miR-340 is responsible for a high level of ABCB5 and other proteins under hypoxic conditions [31]. In 2019, Martinez-Usatorre et al. reported that expression of miR-155 depends on the presence of CD8^+^ T cells in tumour infiltrating lymph nodes, and low levels of miR-155 are associated with longer overall survival (OS) in melanoma patients [28]. These results indicate that miRNAs are key regulators of T cell function in the TME.

### 2.3. Role of miRNA in Regulating Tumour-Associated Macrophages (TAMs)

TAMs have two faces, pro- and anti-tumourigenic, depending on the classical M1 or M2 subtype. Macrophages of the M2 phenotype create an immunosuppressive microenvironment that favours tumour progression. Infiltrating M2 macrophages are observed mostly at the late stage of human cancers. In lung cancer, an increased miR-21-5p level promotes the polarisation of the M2 phenotype [32]. In contrast, miR-125b binds to and inhibits tumour necrosis factor-α (TNF-α), maintaining the M1 phenotype [33]. MiRNA can also regulate other functionalities of TAMs, including infiltration, immune responses and the tumour-promoting process. Another crucial miRNA involved in the modulation of immune responses is miR-155, which is downregulated in TAMs [34]. Squadrito et al. (2012) reported that miR-511-3p has a regulatory effect on TAMs, suggesting that overexpression of miR-511-3p in TAMs can block tumour growth [35]. Released from tumour cells, miR-21 and miR-29a reach macrophages via microvesicles and bind to intracellular Toll-like receptors (TLR7 or TLR8). This increases the production of pro-inflammatory pro-tumour cytokines (IL-6 and TNF-α) in TAMs by activating the NF-κB pathway [36]. It was shown that melanoma exosomes induce macrophages to a tumour-promoting TAM phenotype. The authors showed that, in melanoma, miR-125b-5p is delivered by exosomes to macrophages, which supports the tumour-promoting TAM phenotype. Therefore, the miRNA of the TME play a key role in regulating the phenotype and function of TAMs through several pathways.

### 2.4. Role of miRNA in Regulating Tumour-Associated MSCs

Mesenchymal stem cells (MSCs), also known as mesenchymal stromal cells, participate in normal stroma formation as well as tumour stroma formation contributing to tumour progression. Bone marrow-derived MSCs (BM-MSCs) can migrate to tumour niches, where they can promote pro-metastatic effects. Zhu et al. demonstrated that downregulation of miR-155-5p induced BM-MSCs to activation of nuclear factor-κB p65 (NF-κB p65) in gastric cancer [37]. The overexpression of miRNA suppresses the metastasis-promoting activity of cancer-associated MSCs in many types of cancer, such as prostate, multiple myeloma and myeloid neoplasms. This evidence indicates that the abnormal expression of miRNA can influence MSCs in the TME, activating tumour growth and progression. To our knowledge, no association has been found so far between miRNA effects on tumour-associated MSCs as part of the TME in melanoma cancer, which is probably only a matter of time.

### 2.5. Role of miRNA in Regulating Endothelial Cells

MicroRNAs can play a role in the cell where they are produced, influence the phenotype of nearby melanoma cells or execute their function in the tumour microenvironment by affecting ECs. Melanoma cells express high levels of miR-1908, miR-199a-5p and miR-199a-3p. As a result, they have a greater capacity to recruit ECs in vitro and in vivo [38]. Melanoma cell-derived exosomes promote the activity and migration of ECs. By transferring miR-9 to ECs, Zuang et al. (2012) activated the JAK-STAT pathway and stimulated angiogenesis [39]. In fact, miRNA acts on the angiogenesis process by also influencing vascular endothelial growth factor C (VEGF-C) and platelet-derived growth factor C (PDGF-C). For example, miR-214 induces MET expression, which is associated with the high expression of VEGF-C and PDGF-C and enhanced lymphangiogenesis. VEGF-C activates the proliferation of lymphatic ECs, and PDGF-C, as a mitogenic factor and chemoattractant, activates not only CAFs but also blood ECs [40]. These results demonstrate that miRNAs are crucial for tumour angiogenesis by regulating EC activity and VEGF signalling. Torii et al. (2021) investigated soluble factors secreted from highly metastatic tumours [41]. They observed that a high level of miR-1246 was present in highly metastatic tumours. MiR-1246 was transported via extracellular vesicles into ECs and induced IL-6 expression. These results suggest that highly metastatic tumours induce drug resistance in ECs by transporting miR-1246.

## 3. Role of miRNA in Regulating Tumour Progression

**Figure 1:**
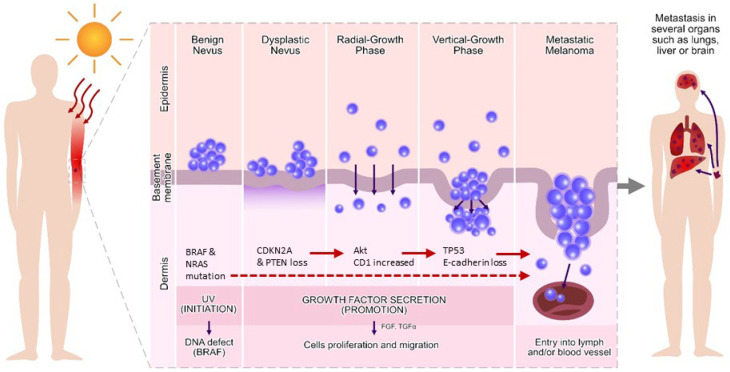
Process of metastasis in melanoma skin cancer and changes at the molecular level from initiation to progression of melanoma. This illustration depicts melanoma progression and molecular alterations that can occur at different stages, from benign nevus to metastatic melanoma. BRAF and N-RAS are commonly mutated in melanoma, as week as the heritable mutation (CDKN2A), but germline mutations (PTEN and TP53) develop melanoma rarely. Spontaneous DNA mutations in several genes involved in the progression of melanoma are encountered at different stages of the disease.

The deregulation of miRNA has been described for almost every aspect of cancer formation and progression [42]. MicroRNA can act as onco-miRNA or tumour suppressive-miRNA, and their regulation is associated with several hallmarks of melanoma pathogenesis (Figure 1), such as the promotion of proliferative signalling (e.g., miR-137, miR-221), resistance to cell death (miR-18b and miR26a), replicative immortality (e.g., miR-205, miR-203) and invasion or metastasis (miR-214, miR-31) [43]. The data on roles of various miRNA in melanomagenesis were compiled and are presented in Table 1. This part of the manuscript presents knowledge of miRNA dysregulation in melanoma progression.

### 3.1. Important Biochemical and Signalling Pathways in Melanoma

Melanoma originates from neural-crest-derived melanocytes (Figure 1). Melanin production starts in melanosomes during a biochemical process known as melanogenesis [57]. The transformation of melanocytes into melanoma cells is a multi-step process that changes concentrations of growth factors, cytokines, nutrients, glucose and oxygen [58]. Melanocytes produce cytokines, pro-opiomelanocortin (POMC), nitric oxide (NO), prostaglandins and leukotrienes, involved in inflammatory responses [59]. Biochemical pathways, essential for tumourigenesis, are regulated by epigenetic phenomena, e.g., remodelling of the nucleosome by histone modifications, DNA methylation and miRNA [60].

The most important mutation in melanomas—BRAF—is detected in 66% of malignant melanomas [61]. In general, melanomas carry a mutated NRAS, BRAF or concurrent BRAF and PTEN mutations [62]. About 80% of benign nevi carry BRAF [63] but none in familial melanomas [64]. In addition to BRAF, melanomas have the activity of many signalling pathways related to oncogenesis, including PI3/Akt kinase, NFB, Src and STAT3. Other mutations include CDNK2A deletions, MITF amplification and PTEN cleavage activating PI3/Akt kinase [65]. The PTEN is also an important element altered in signal transduction in melanomas. Its downregulation limits apoptosis, and strong expression of PTEN induces apoptosis by inhibiting the protein kinase B/Akt phosphorylation [66]. The most commonly mutated oncogenes in melanomas are the RAS genes. They control important biochemical processes and apoptosis through the PI3K-PTEN-Akt pathway. The best described pathway for RAS is the receptor tyrosine kinase-MAPK [67], which includes BRAF and controls cell proliferation by RAS, mainly tumour cell proliferation by activated RAS. This results in phosphorylation of Akt and protection against apoptosis. These genes are often mutated in melanomas, so the RAS-PTEN/BRAF axis is mostly abnormal. Genetic changes in BRAF and RAS result in hyperactivation of the RAS/RAF/MEK/ERK (MAPK) signalling pathway in most melanomas. The pathogenesis of melanoma also indicates the involvement of WNT and PI3K signalling pathways, which play a major role in the progression of melanoma [57]. The RAS/RAF/MEK/ERK signalling pathways determine the resistance of melanoma cells to inhibitors used in immunotherapy, which is based on the blocking the checkpoints programmed death-1 (PD-1) or cytotoxic T-lymphocyte-associated protein 4 (CTLA4) by antibodies. Both checkpoints are physiological controllers of the T-cell-mediated immune response [68]. Extra- and intracellular factors control melanin biosynthesis (melanogenesis) and act through various intracellular signalling pathways, ultimately leading to regulation of a key transcription factor—MITF (associated with microphthalmia)—and expression of TYR, TYRP-1 and TYRP-2 melanogenic enzymes.

Various epigenetic factors are involved in the regulation of melanogenesis [69], including miRNAs, e.g., miR-340, miR-218 and miR-137, which become tumour suppressors by downregulation of the MITF [70,71]. MiRNAs can modulate the expression of genes encoding extracellular mediators (wnt3a), membrane receptors (IGF-1R), intracellular components of melanogenic signalling pathways (SOX transcription factors) or molecules involved in melanosome transfer (Myo5a, Rab27a and Fscn1) [72,73].

### 3.2. MiRNA in Tumour Growth

Tumour growth is conditioned by communication between cancer cells and TME cells, including ECs, Fbs, immune cells (e.g., T cells and macrophages), TAMs and CAFs. Fibroblasts surround the growing tumour mass, forming a framework in their early development. Recent studies [74] have shown that high levels of miR-7 can inhibit RAS-association domain family member 2 expression in CAFs, which enhances cell proliferation. Increased levels of miR-27a inhibit the differentiation of Th1 and Th17 cells by the action of dendritic cells and enhance the growth of melanoma cells [75]. This suggests that TME-associated miRNAs play a key role in melanoma tumour growth. It has also been reported that miR-101 and miR-3157 negatively regulate the EZH2 gene and promote cancer progression [45]. It has been shown by Dror et al. (2016) that melanoma cell-derived miR-211 induces CAF formation by targeting insulin-like growth factor 2 and activating the MAPK signalling pathway, which in turn enhances the growth of melanoma [22]. In 2019, Wang et al. also published results showing that the loss of CAF-derived exosomal miR-3188 may affect the proliferation and apoptosis of tumour cells [76].

### 3.3. MiRNA in Tumour Angiogenesis

Angiogenesis is the process of capillary/vessel formation. It occurs during embryonic development and may also occur in postnatal life. Forming new vessels via angiogenesis, intravasation and extravasation is an important step during melanoma progression. Due to angiogenesis, cells from the primary tumour can spread through the entire body through the vascular vessels and form secondary tumour mass in distant tissues/organs.

Pencheva et al. (2012) has described the involvement of miR-1908, miR-199a-5p and miR-199a-3p in angiogenesis during melanoma progression [38]. MiR-203 has been shown to suppress the migration of melanoma cells and angiogenesis in an in vitro study. This resulted in limiting the growth of the primary tumour and inhibiting metastasis to the lungs and lymph nodes [77]. The involvement of miR-214 in extravasation has been noticed both in vivo and in vitro [49]. In vitro study showed that trans-endothelial migration of miR-214 was typical for cells with high levels of miR-214 in comparison to the low miR-214 expression of the A375P cell line (described as poorly metastatic). In vivo, extravasation of miR-214-overexpressing cells from blood vessels stimulated the formation of distant metastasis (e.g., into lungs). Moreover, miR-214 has been shown to reduce anoikis, a type of apoptosis caused by loss of connectivity with an extracellular substance or with other cells, which could be important for melanoma cell survival in the blood circulation.

### 3.4. MiRNA in Tumour Metastasis

The crosstalk between the matrix and cancer cells decides whether cancer cells remain settled or become invasive and metastatic tumours. The phenomenon of metastasis is responsible for approximately ~90% of cancer-related deaths. It is a key element in cancer staging systems, such as tissue–lymph nodes–metastasis (TNM). The metastatic ability of tumour cells is enhanced by their interactions with tumour stromal cells in the TME. There have been several studies showing the influence of miRNA on the process of melanoma metastasis.

MiRNA can participate in several steps of the metastatic process. Low levels of CAF-derived exosomal miR-148b transferred to endometrial cancer cells increased the amount of cancer metastasis. This depended on DNA-methyltransferase 1, which is an important regulator of cancer metastasis [78]. Low expression of miR-203 was observed in metastatic melanoma compared to primary melanoma, and hypermethylation of the miR-203 promoter has been suggested as a potential mechanism of tumour metastasis. Reduction in IL-8 levels by miR-203 can play an important role in the neovascularisation process [77].

### 3.5. MiRNA in Drug Resistance

Drug resistance is a crucial step affecting patient prognosis. Combination chemotherapy has become a new paradigm for cancer therapy that has led to the development of increasingly complex regimens. CAFs in the TME are closely associated with chemoresistance acquisition and a poor clinical prognosis. Dysregulation of miRNA molecules in CAFs results in drug resistance, as well as the transfer of exosomal miRNA between cancer cells and the TME, which is correlated with chemoresistance regulation.

Therapeutics such as BRAFV600 provide hope for patients with metastatic melanoma. Several mechanisms involve miRNA in the development of melanoma resistance to BRAF inhibitors (BRAFi) and MEK inhibitors (MEKi), which block the MAPK pathway; this limits their prolonged use [79,80,81]. It has been demonstrated by Liu et al. (2015) that a low level of miR-200c correlates with the development of resistance to BRAFi, based on clinical samples [51]. In an in vitro study based on resistant melanoma cells and tumour biopsies from treated patients, Vergani et al. (2016) detected high expression of miR-100 and miR-125b [82]. Expression of miR-579 in melanoma cells can revert drug resistance to BRAFi and, more interestingly, prevent the development of acquired drug resistance. The second therapy associated with miRNA in melanoma is treatment with immune checkpoint inhibitors (ICIs) [83,84]. Circulating miRNAs such as let-7e, miR-99b, miR-100, miR-125a, miR-125b, miR-146a, miR-146b and miR-155 are associated with the activity of myeloid-derived suppressor cells (MDSCs) and have been identified in melanoma patient samples [83]. These miRNAs are involved in polarisation and differentiation of myeloid cells and responsible for the conversion of monocyte cells into MDSCs. Their levels correlate with the clinical efficacy of ICI, which is why miRNAs are being considered as new therapeutic strategies to overcome BRAFi and ICI resistance.

## 4. MiRNA in the Immune Response to Cancer

Immune cells infiltrate the TME to induce tumour cell killing and may be regulated by miRNA or its target genes present in the TME [85]. Similarly, miRNA found in exosomes, which are extracellular vesicles found in body fluids, can affect the TME [86]. Additionally, exosomal miRNA have the ability to influence the function of immune factors and cells. This is particularly important because it has been shown that exosomal miRNA can bind to Toll-like receptors (TLRs) in non-small cell lung cancer (NSCLC) and affect tumour growth and metastasis and in pancreatic adenocarcinoma with TLR4 with similar results. Exosomal miRNAs also affect tumour proliferation and act as positive mediators of angiogenesis [86]. MiRNAs are able to regulate macrophage polarisation, including TAMs, which are associated with inflammation in the TME. TAMs are classified as alternatively activated macrophages (M2) [87]. Zhao et al. (2021) reported a correlation between low expression of LIF receptor subunit alpha (LIFR) and increased immune cell infiltration in the TME in oesophageal cancer (ECs) [85]. LIFR is one of seven miRNA genes associated with poor patient prognosis [85]. MiRNAs, together with transcription factors, collectively regulate the expression of immunomodulators in various types of carcinomas [88]. An effect of miRNA on the expression of and interaction between programmed death-ligand-1 (PD-L1) and programmed cell death protein-1 (PD-1) has also been reported. Thus, miRNA modulates T lymphocyte function in cancer patients. Along with this function, miRNA regulates CTLA-4, induces epithelial-mesenchymal transition (EMT), affects the tumour immune response and suppresses EMT by inhibiting ZEB1 and ZEB2 [89]. MiRNAs also regulate the expression of immune checkpoints such as LAG-3, TIM-3, BTLA and CTLA-4 [89]. MiR-155, a multifunctional miRNA, is involved in a number of activities that modify the immune response in tumour patients: it affects tumour cell proliferation, downregulates the expression of the immune response modulator SHIP1 resulting in induction of pro-inflammatory cytokine production, participates in the impaired development of immune cells (B and T lymphocytes, dendritic cells) and impairs their function (macrophages, mast cells) [90,91]. When miR-155 is deficient in TAMs, it tends to convert from an M1 to an M2 phenotype, which is pro-tumour and anti-inflammatory [92]. The miR-155-induced transformation of dendritic cells from immunosuppressive cells to immunostimulatory cells with the ability to induce a strong immune response towards the tumour has been demonstrated [93]. It is likely that such high activity of this miRNA is due to the targeting and degradation of inflammation-related genes [91]. Because of its multifunctional activities, miR-155 has been investigated for a therapeutic role with positive results in the form of therapies focusing on regulating miR-155 expression [90]. A potential therapeutic role is also indicated by the ability of miR-155 to modulate and inhibit the expression of PDL-1 which, together with PD-1, forms a complex used to treat malignancies [91]. MiR-21, an oncogenic miRNA found in most cancers, plays a major role in Sézary disease, where its expression in cancerous CD4 memory T cells is elevated relative to CD4 T cells in healthy patients, and its activity is associated with signal transducer and activator of transcription 3 (STAT3) [94]. MiR-30b/d affects T cell recruitment by decreasing it and Tregs by increasing their induction via the regulation of IL-10 secretion [92]. It was shown that miR-17-92 expression is downregulated in mouse T cells, causing them to have inferior antitumour activity [95]. The development of autoimmunity and lymphoproliferative disease in mice with lymphocytes with elevated miR-17-92 expression in vivo was observed [96].

Cancer patients were categorised by Thorsson et al. (2018) into six immune infiltration subtypes (C1-6) characteristic of different cancer types (Table 2) and representative features of the TME [88]. The first subtype, C1 (wound healing), is characterised by high proliferation rates, deviation of Th2 cells to adaptive immune infiltration, and elevated expression of angiogenic genes. The second C2 (INF-r dominant) subtype is also characterised by a high proliferation index but also by the highest M1/M2 macrophage polarisation ratio, high TCR diversity and strong CD8 signalling. The CD3 (inflammatory) subtype shows elevated Th17 and Th1 genes, lower levels of aneuploidy and overall somatic copy number changes than the other subtypes and low to moderate tumour cell proliferation. The C4 (lymphocyte depleted) subtype shows a prominent macrophage signature, with a high M2 response and Th1 suppression. Subtype C5 (immunologically quiet) is characterised by the lowest level of lymphocytes and the highest level of macrophages, with M2 predominance. Subtype C6 (TGF-β dominant) shows high lymphocyte infiltration with an even distribution of Th1 and Th2 cells and the highest TGF-β signature [88].

## 5. Hypoxic and Normoxic Profiles of miRNA in Melanoma

The hypoxic environment in melanoma is responsible for the initiation of metastasis through the induction of major metastatic cascades, the suppressive role of miR-205, melanoma cell proliferation (miR-205, E2F1, miR-211), increased migration and invasion of melanoma cells (miR-1290, miR-211), phenotypic plasticity, drug resistance and increased cellular heterogeneity [97,98,99,100].

Tittarelli et al. (2020) have reported that, in studies on human and mouse melanoma cell lines, in hypoxic melanoma cells, HIF-1α binds the GJA1 promoter and induces the expression of the gap junction protein connexin-43 (Cx43) [27]. Hypoxia disrupts Cx43-gap junction intercellular communication (GJIC) and reduces the susceptibility of melanoma cells to cytotoxic attack by natural killer cells and allows them to establish more GJIC with dendric cells and melanoma-specific cytotoxic T lymphocytes (CTLs) [27]. Hypoxia-induced miR-210 has the ability to lyse CTLs and to reduce the susceptibility of tumour cells to lysis by T cells. The regulatory activity of miR-210 has a positive effect on melanoma cell growth, even under anaerobic conditions [100].

Wozniak et al. [97], by analysing miRNA upregulated from exosomes, showed that they affect multiple functions such as cell–cell interactions and TME modification, are involved in the control of proliferation and drug resistance (MAPK, ErbB and RAS signalling) and regulate specific properties of tumour-initiating cells and abnormal protein folding. Important roles also include favouring a metastatic phenotype and facilitating tumour invasion by exosomal miR-1246 and miR-6088 [97]. In a study on canine oral malignant melanoma (COM), which may be a good model for human melanomas such as human oral mucosal melanomas, it was shown that, depending on the cell line, i.e., primary (KMeC) or metastatic (LMeC), the expression of the hypoxia miRNA profile differed [98]. The hypoxic state in melanoma increases the secretion of small extracellular vesicles into the TME, resulting in an increase in total proteins compared to normoxia [99].

## 6. Expression Profile of miRNA in Melanoma and Its Progression

Studies have shown that high-frequency copy number abnormalities occur in genome-wide miRNA-containing regions in many human epithelial cancers. Copy number alterations in melanoma samples are correlated with miRNA expression. Zhang and co-workers showed that copy number changes in genes contributed to the biogenesis and functionality of miRNA in cancer samples [101]. In a study comparing miRNA expression in melanoma cancer cells with that in healthy skin, differences in the expression of 143 miRNAs were observed. Microarray analysis showed that the expression of 32 miRNAs increased, while 111 decreased [102]. The deregulated miRNAs with increased expression in melanoma samples included miR-146a-5p, miR-4454, miR-7975, miR-17-5p, miR-29a-3p, miR-30d-5p and miR-20a-5p. They are suggested to be involved in the regulation of TLR, NF-κB and ErB receptor-related signalling pathways [102,103]. However, upregulated miR-138-5p, miR-146b-5p, miR-664b-3p, miR-146a-5p and miR-509-3-5p and downregulated miR-877-3p, miR-4300, miR-4720-3p and miR-6761-5p showed the greatest differences in expression. They have been shown to be involved in 28 different signalling pathways, most of them involved in carcinogenesis [102]. Another study comparing normal human epidermal melanocytes (NHEMs) with melanoma cell lines studied the expression of 461 miRNAs. Deregulation of miRNA expression was considered when the decrease or increase in expression was at least threefold. There were 91 miRNAs meeting this criterion, of which 77 showed higher expression and 14 showed lower expression in primary melanoma cell lines. To highlight only those highly upregulated miRNAs, the list was narrowed down to those with 10-fold difference in expression and those that were not expressed in NHEMs. Upregulated miRNAs include miR-18a*, miR-504, miR-640, miR-196b, miR-507, miR-641, miR-200a*, miR-517*, miR-658, miR-200c, miR-518a, miR-662, miR-218, miR-518f*, miR-758, miR-26b, miR-520b, miR-9, miR-27b, miR-520d, miR-92b, miR-301, miR-520d*, miR-30a-3p and miR-525, while downregulated miRNAs include: miR-148b, miR-331, miR-489, miR-181a, miR-363, miR-503, miR-23b, miR-422b, miR-527, miR-299-3p, miR-455, miR-595, miR-324-5p and miR-485-3p [104]. Analysing the above data, it can be observed that the results do not overlap, and further studies are worthwhile in the context of the expression profile of miRNA associated with early melanoma progression.

To identify miRNA associated with metastatic colonisation, miRNA expression levels were compared between primary melanoma cell lines and melanoma cell lines derived from metastatic melanomas. To be considered deregulated, individual miRNAs had to meet the following criteria: (I) at least threefold deregulation; (II) at least 20% of average signal fluorescence intensity; (III) miRNA expressed in all melanoma cell lines and (IV) miRNA considered deregulated in metastasis had to be expressed at the same level in melanocytes and primary melanoma cell lines. The authors demonstrated 13 miRNAs in metastatic melanoma cell lines that were deregulated. Among them, 11 were upregulated: miR-199a*, miR-515-3p, miR-519d, miR-302c*, miR-517b, miR-520f, miR-30a-5p, miR-518b, miR-523, miR-425-3p and miR-519b. Only two miRNAs were downregulated: miR-30e-3p and miR-514 [104].

To obtain data on the role of miRNAs in melanoma development, numerous profiles of their expression in both NHEMs and melanoma cells derived from primary or metastatic cells have been performed [103]. It was found that miRNA such as miR-133a, miR-199b, miR-453, miR-520f, miR-521 and miR-551b are upregulated both during tumour development from healthy melanocytes to primary cancer cell and from primary cell to metastatic melanoma. They were upregulated threefold higher in expression compared to healthy tissue. Furthermore, miR-190 expression was significantly reduced in primary and metastatic melanoma cells compared to melanocytes [104]. An initial increase in expression was observed for miR-126, miR-29c, miR-506, miR-507 and miR-520d*, but this decreased in the metastatic phase [103]. Another study by Levati and co-workers examined the expression of miRNA (miR-17-5p, miR-18a, miR-20a, miR-92a, miR-146a, miR-146b and miR-155) by quantitative real-time PCR. The selection of individual miRNAs was based on their previous studies on cancer development and progression. Melanocyte cultures and melanoma cell lines were used in the experiment. The authors showed that miR-17-5p, miR-18a, miR-20a and miR-92a were overexpressed, while miR-146a, miR-146b and miR-155 were downregulated in most melanoma cell lines. Furthermore, they confirmed that ectopic expression of miR-155 significantly inhibited the proliferation of melanoma cell lines and induced their apoptosis [105].

## 7. Conclusions

MiRNA molecules are involved in all stages of melanoma metastasis, such as intravasation into the lumen of vessels, survival in the cardiovascular or lymphatic systems, extravasation, and formation of the pre-metastatic niche in distant organs, such as the lungs, brain and liver. Melanoma progression is provided by metabolic alterations in which miRNA strongly participates. Evidence demonstrates that miRNA expression is dysregulated in numerous cancer types and that miRNA expression profiles are capable of classifying human tumours, which can be correlated with clinical outcomes in melanoma cancer patients. This is very important because targeted miRNA therapies can influence the development and course of melanoma, as well as increase sensitivity to conventional therapies and immunotherapies.

## Figures and Tables

**Table 1:** MiRNA affected progression in melanoma.

Hallmark of Cancer	↓ or ↑ Regulation	miRNA	Reference
Changing the methylation of DNA	↓	miR-29	[44]
Cell differentiation	↓ ↑	miR-201 miR-449a	[45,46]
Cell cycle	↓ ↑	miR-101 miR-449a	[45,46]
Resisting cell death	↓ ↑	miR-18b, miR-26a, miR-137, miR-193b, miR-205 miR-290–295	[47]
Cell proliferation	↓ ↑	miR-99a, miR-101 miR-200c	[45,48]
Cell migration	↓ ↑	miR-31, miR-101 miR-200c	[45,48]
Invasion	↓ ↑	miR-31, miR-101, miR-9, miR-137, miR-148, miR-182 miR-214	[45,49,50]
Inducing angiogenesis	↑	miR-1908, miR-199a-5p, miR-199a-3p	[43]
Drug resistance	↑	miR-200c	[51]
Maintaining proliferative signalling	↓ ↑	miR-10, miR-22, miR-26a, miR-30, miR-34, miR-125a, miR-137, miR-193b, miR-205, miR-211 miR-221, miR-15b, miR-149, miR-7, miR-506–514, miR-137, miR-17-19, miR-92a, miR-222	[43,52,53,54,55,56]

**Table 2:** Immune infiltration type vs. cancer type [88].

Immune Infiltration Subtype	Cancer Type
C1	colorectal cancer: COAD (colon adenocarcinoma) READ (rectum adenocarcinoma) lung squamous cell carcinoma (LUSC) breast invasive carcinoma (BRCA) luminal A head and neck squamous cell carcinoma (HNSC) chromosomally unstable (CIN) gastrointestinal subtype
C2	highly mutated BRCA gastric tumours ovarian (OV) tumours head and neck squamous cell carcinoma (HNSC) cervical tumours (CESCs)
C3	kidney carcinoma prostate adenocarcinoma (PRAD) pancreatic adenocarcinoma (PAAD) papillary thyroid carcinomas (THCAs)
C4	adrenocortical carcinoma (ACC) pheochromocytoma and paraganglioma (PCPG) liver hepatocellular carcinoma (LIHC) gliomas
C5	brain lower-grade gliomas (LGGs)
C6	mixed tumours not dominant in any one subtype
